# Volumizing Threads (Jamber) in the Midface and Managing Side Effects: Clinical Cases

**DOI:** 10.1055/a-2303-5156

**Published:** 2024-06-14

**Authors:** Kyu-Ho Yi, Soo-Yeon Park

**Affiliations:** 1 Division in Anatomy and Developmental Biology, Department of Oral Biology, Human Identification Research Institute, BK21 FOUR Project, Yonsei University College of Dentistry, Seodaemun-gu, Seoul, The Republic of Korea; 2 Maylin Clinic (Apgujeong), Seoul, (the Republic of) Korea; 3 Division of Aesthetic Made-Young Plastic Surgery Clinic, Seoul, The Republic of Korea

**Keywords:** jamber thread, volumizing thread, midface rejuvenation, thread lifting

## Abstract

The clinical application of polydioxanone (PDO) threads, traditionally utilized for tissue lifting, is now being explored for its volumizing effects in midface rejuvenation. The novel approach involves employing PDO volumizing threads to achieve physical augmentation akin to a “solid filler.” The study introduces a more convenient insertion method for these threads, prioritizing ease and efficacy. Clinical cases demonstrate the efficacy of volumizing threads in addressing midface concerns, such as nasolabial folds and midcheek grooves. Additionally, the integration of volumizing threads to provide support in sagging areas is examined for achieving natural-looking enhancements. While highlighting positive outcomes, potential side effects like thread protrusion are addressed, along with strategies for their mitigation. Volumizing threads are presented as a suitable procedure for patients wary of traditional fillers or seeking subtle enhancements, with the recommendation of combining them with cog threads for those desiring more pronounced changes in facial contour. In summary, volumizing thread offers a minimally invasive alternative with fewer side effects for midface rejuvenation.

## Introduction

As individuals age, prominent manifestations include a decline in skin elasticity and a reduction in facial volume, attributed to diminished collagen synthesis and the gravitational effects on facial tissues, resulting in facial ptosis. (The Facial Aging Process From the “Inside Out”) While cog thread lifting, a technique aimed at facial tightening, has been extensively investigated, 1 2 3 4 there exists a relative scarcity of literature regarding volumizing threads dedicated to enhancing facial volume, particularly when contrasted with conventional lifting approaches. Nevertheless, the clinical relevance of volumizing threads remains significant. 5

The significance of polydioxanone (PDO) threads in aesthetic procedures is well-established. Widely recognized for their tissue-lifting properties, PDO threads are extensively utilized in clinical settings. These threads serve multiple purposes, including the improvement of static wrinkles and addressing areas requiring volumization, making them a preferred option, especially for patients reluctant to undergo hyaluronic acid (HA) filler injections. 6 7 8

PDO threads demonstrate strong tensile strength compared with other absorbable threads and have a dissolution period exceeding 6 months. This prolonged duration of effectiveness contributes to their widespread adoption. (What are the Factors that Enable Thread Lifting to Last Longer?, Tissue changes over time after polydioxanone thread insertion: An animal study with pigs, Histological and molecular biological analysis on the reaction of absorbable thread; Polydioxanone and polycaprolactone in rat model) Additionally, despite having a lower elastic limit, PDO threads possess a high elastic modulus, providing robust elasticity due to material rigidity. (Two years' outcome of thread lifting with absorbable barbed PDO threads: Innovative score for objective and subjective assessment., Use of Polydioxanone Threads as an Alternative in Nonsurgical Procedures in Facial Rejuvenation) This firmness offers adequate support to tissues during the initial stages of the procedure and helps maintain lifted skin and tissues to some extent, even during facial movements. (What are the Factors that Enable Thread Lifting to Last Longer?)

**Fig. 1:**
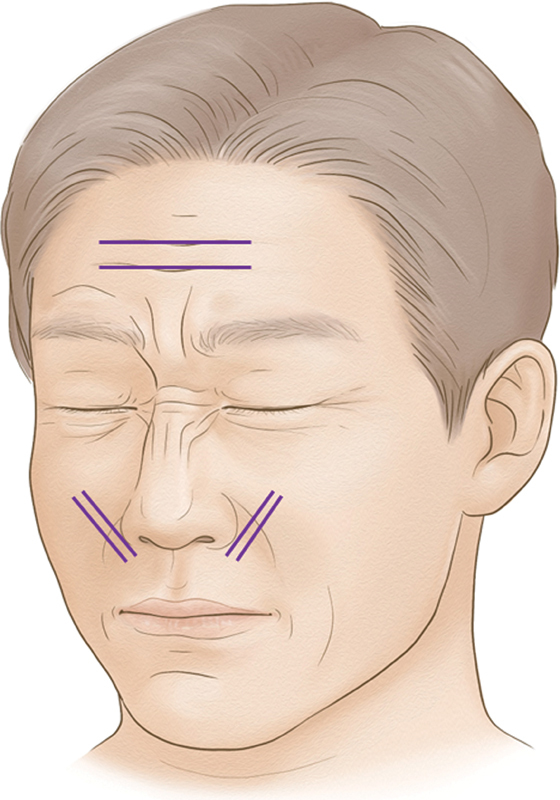
Demonstrating the use of the conventional polydioxanone cog thread, folded and inserted directly beneath the static wrinkle, resulting in a volumizing effect.

When employing conventional PDO cog thread as depicted in Fig. 1 , they can be strategically folded and inserted directly beneath static wrinkles to achieve a volumizing effect. This thread augmentation process yields physical volume augmentation, later complemented by mild local reactions such as edema, lymphocytic infiltration, and fibrosis. Referred to as a “solid filler” in the literature, PDO threads serve as a robust alternative to soft tissue fillers 9 ; however, the insertion process for cog threads can be cumbersome due to the need for rotation and withdrawal. To address this, exploration into PDO cog volumizing threads in this study proposes a more convenient insertion method, involving simultaneous use of multiple threads without overlapping.

**Fig. 2:**
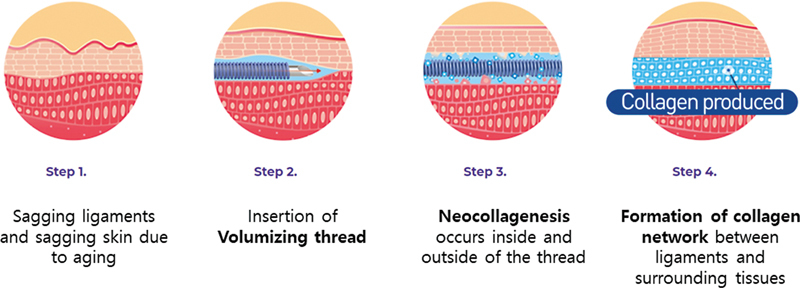
Demonstrating the process of tightly winding a long thread similar to a rubber band, effectively compressing the thread to increase volume. Abundant collagen forms inside and around the thread, creating a network between the reinforced fibrous tissue and the surrounding area.

**Fig. 3:**
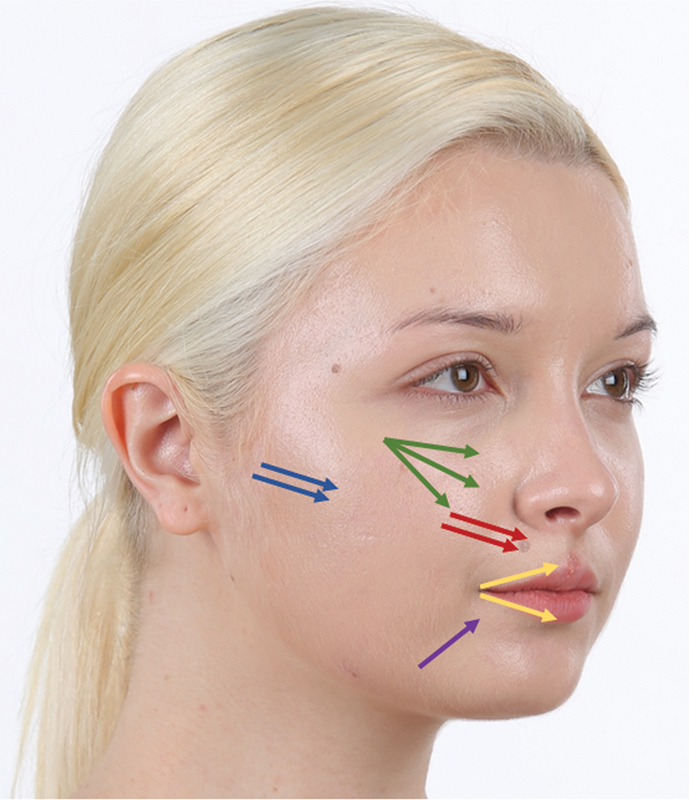
Example of Jamber thread insertion on the anterior cheek (green arrow), nasolabial fold (red), subzygomatic arch depression (blue arrow), vermilion line (yellow), and prejowl sulcus (purple).

This innovative technique utilizes tightly wound long threads, similar to a rubber band, to achieve a convenient and effective method of thread compression for volumetric enhancement. Abundant collagen deposition occurs both inside and around the thread, facilitating the formation of a network between reinforced fibrous tissue and the surrounding tissue through the generated collagen ( Fig. 2 ). Furthermore, the thread itself, with its thickness, provides stability and a natural outcome to the deflated area, supporting and lifting the sagging region. When utilized alongside fillers, the spring-shaped scaffold within the thread partially prevents filler migration, enabling the creation of the desired voluminous shape precisely in the targeted location ( Fig. 3 ).

## Volumizing Thread (Jamber Thread)

**Fig. 4:**
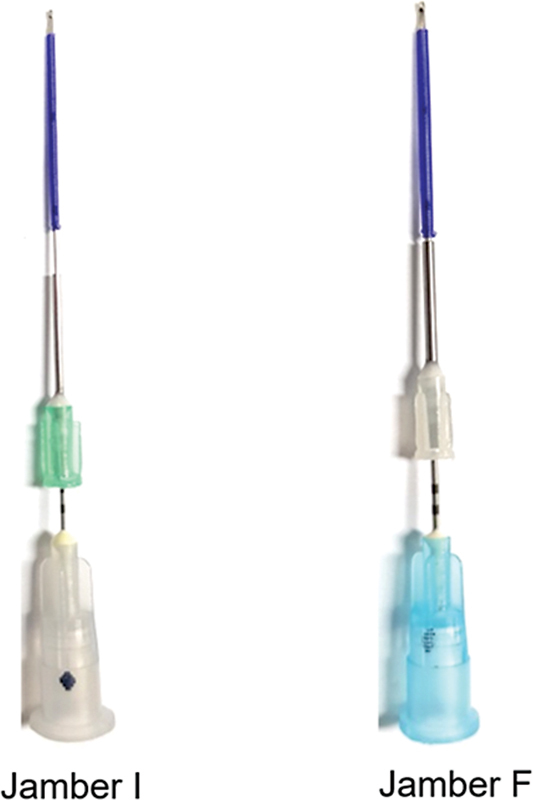
Jamber thread, a volumizing thread, available in various sizes including Jamber I (27G) and Jamber F (21G, 23G), providing flexibility in selection.

In essence, the introduction of volumizing thread (Licellvi Jamber F [JWorld Co., Ltd.] and countourel Jamber F [Croma Pharma GmbH], Fig. 4 ) serves to stabilize the sagging area while concurrently administering filler in that specific location. This approach allows for precise volumizing effects without filler displacement, particularly in regions such as marionette lines or nasolabial folds. The maintained and undistorted appearance can be attributed to the effective action of the thread, which penetrates the skin, smoothing wrinkled lines, enhancing volume, and preventing volume loss through the promotion of collagen regeneration in high-tension areas.

**Fig. 5:**
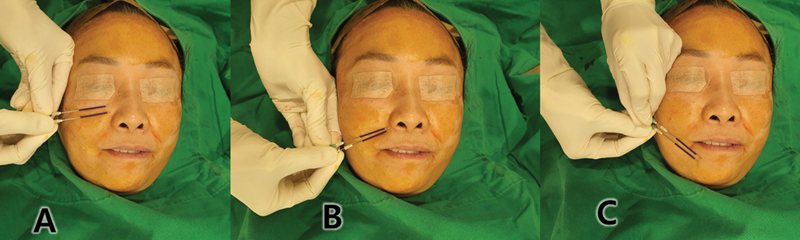
To correct the Indian band, a thread is gently inserted, extending slightly beyond the targeted area ( **A** ). Similarly, in addressing marionette lines ( **B** ), the thread is inserted to subtly modify the fold, reducing the volume difference between the adjacent areas. Additionally, a slightly more acute angle is employed within the fold to enhance volumizing effects ( **C** ). Given the relatively superficial insertion depth for marionette lines, a gauge of 27 is commonly utilized.

To address the Indian band, a thread is gently inserted, extending slightly beyond the targeted area, as depicted in Fig. 5A . Similarly, for marionette lines, as shown in Fig. 5B , the thread is inserted to subtly modify the fold, mitigating volume discrepancies between the adjacent areas. Additionally, a slightly more acute angle is utilized within the fold to achieve volumizing effects. Fig. 5C illustrates the application of the same technique to marionette lines. Given their relatively superficial insertion, a gauge of 27 is predominantly used.

Prior to treatment, all participants received comprehensive information regarding the potential benefits and risks of the procedure and provided written informed consent.

### Case 1

**Fig. 6:**
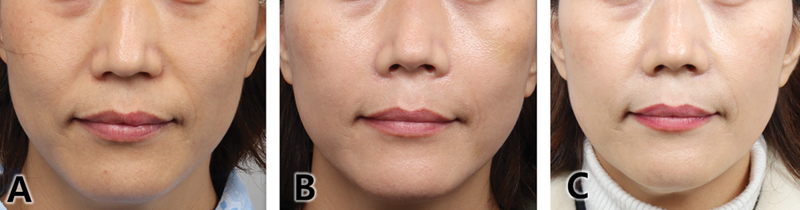
Use of Jamber thread on the nasolabial fold. Improvement of nasolabial folds using 21G Jambe threads, with four lines each, totaling eight lines. Positive results are well-maintained up to 2 weeks ( **A** ), 2 months ( **B** ), and 6 months posttreatment ( **C** ).

A woman in her forties, reluctant to undergo filler treatment yet seeking to improve her nasolabial folds, opted for 21G Jamber threads. Four lines of threads were individually inserted into each nasolabial fold to improve their appearance. Notable improvement was observed by the second week primarily attributable to the volume provided by the coiling threads. Further reduction in the appearance of nasolabial folds and enhanced overall facial contouring was observed during follow-up assessments in the second month ( Fig. 6 ).

### Case 2

**Fig. 7:**
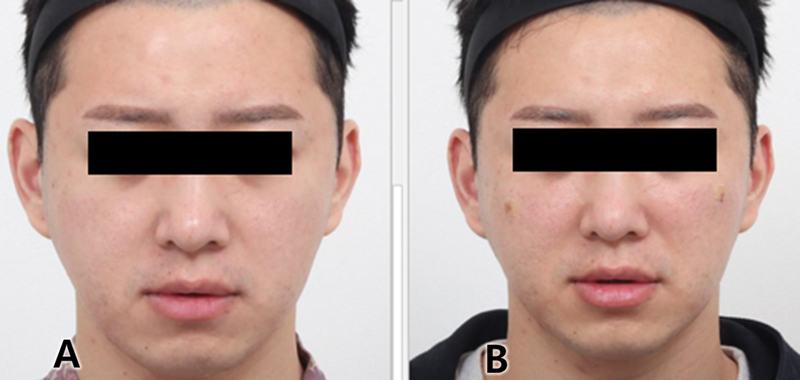
A 31-year-old male with anterior cheek treatment using Jamber 21G, with two threads inserted each side, immediately postprocedure. By inserting Jamber threads through the zygomaticocutaneous ligament, initial volumization is achieved. Subsequently, collagen formation due to fibroblast infiltration around the polydioxanone further enhances the improvement of the midcheek groove. Before ( **A** ) and after 2 months of treatment ( **B** ).

In this case ( Fig. 7 ), a 31-year-old male received 21G Jamber threads, with two sutures on each side, targeting the anterior cheek region. Inserted in a manner traversing the zygomaticocutaneous ligament, the initial impact is due to the volume of the threads. Subsequent improvements in the midcheek groove were observed.

### Case 3

**Fig. 8:**
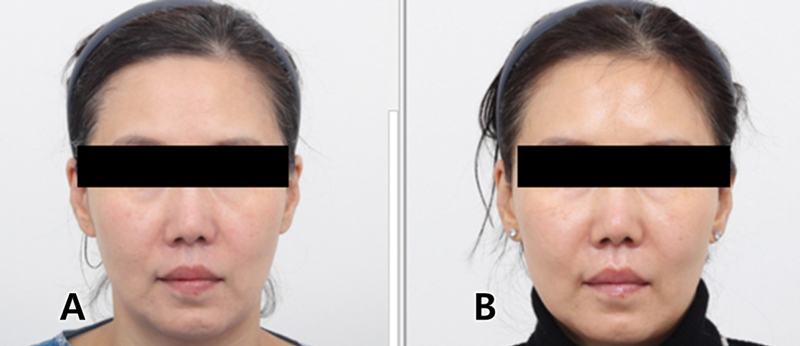
A 50-year-old female with anterior cheek treatment using volumizing two threads each ( **A** ), 6 months posttreatment ( **B** ).

A 50-year-old woman presented concerned with noticeable tear troughs attributed to midface sagging. To address this concern, 21G Jamber threads were inserted into the anterior cheek, with two threads on each side, aiming to enhance volume. Observations revealed well-maintained volume up to approximately 6 months posttreatment ( Fig. 8 ).

All patients reported high satisfaction levels following the application of Jamber threads.

## Discussion

**Fig. 9:**
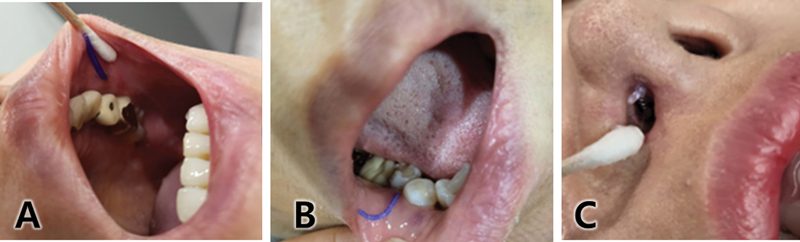
Protrusion of volumizing thread intraorally ( **A, B** ) and intranasally ( **C** ).

The phenomenon can be conceptualized as the formation of a collagen tube, which protrudes in the anteroposterior direction, inducing a lifting effect. Essentially, collagen formation results in the creation of a stick that mimics a ligament, thereby supporting the tissue. 7 Even as the thread dissolves with collagen generation, the induced collagen can help maintain volume. Moreover, when Jamber thread and fillers are used concurrently, the filler occupies the space created by the thread, forming a collagen stick that minimizes filler migration. However, instances may arise where the thickness of the thread causes protrusion ( Fig. 9 ). It may protrude into the oral cavity or around the nasal area, and in such cases, gently pulling on the end can release the thread, causing it to be expelled.

**Fig. 10:**
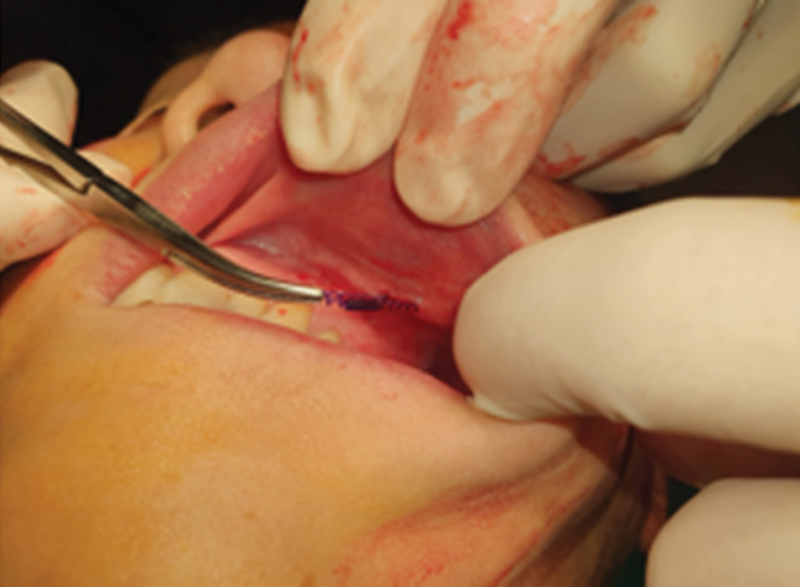
Removal of palpable Jamber near protrusion with the surgical forceps by pulling outward.

**Fig. 11:**
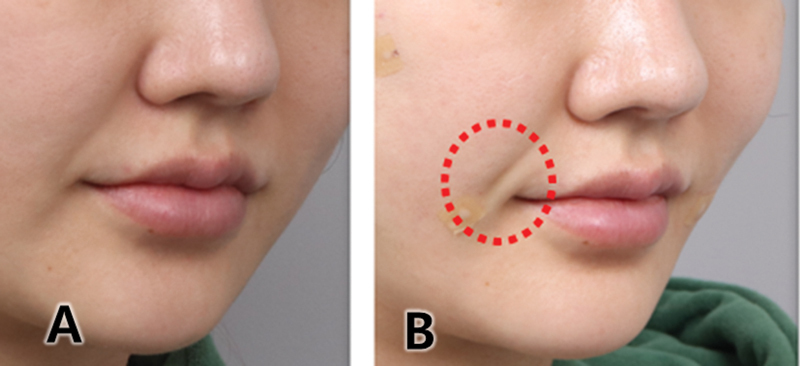
If the Jamber thread is inserted too superficially, there may be cases where the thread touches the dermis, affecting its shape. Panel ( **A** ) is before the treatment and Panel ( **B** ) is the side effect of volumizing thread.

For threads that are on the verge of protrusion, creating a slight hole with a needle and carefully extracting it can facilitate removal ( Fig. 10 ). Additionally, if Jamber thread is inserted too superficially, it may come into contact with the dermis, affecting its shape ( Fig. 11 ). While threads inserted superficially typically improve over time, there is a risk of them becoming persistent scar tissue. Options for improvement include removing the thread through a minor incision or performing subcision and injecting substances such as HA to dissolve the thread.

In a study by Su et al 10 conducted on pigs, PDO threads were found to induce mild inflammation and stimulate collagen production, peaking at 12 weeks and persisting until 48 weeks before complete degradation. Additionally, PDO threads exhibited a fibrous bridging effect, enhancing tissue connectivity and thickness without causing severe side effects such as foreign body granuloma. Furthermore, Ha et al 11 found in a study on rat models that PDO threads significantly increased collagen type 1 α1 after approximately 8 weeks, indicating stimulation of fibroblast proliferation and collagen formation through the TGF-β1 signaling system.

In considering alternative methods for midface lifting, such as fillers or additional mesotherapy, it is essential to weigh the potential benefits against any associated risks. Further investigation into the benefit–harm ratio of these non-Jamber applications can provide valuable insights into their efficacy and safety profiles.

In summary, Jamber threads can provide satisfactory results for individuals with sagging facial areas who may prefer to avoid fillers or those seeking subtle facial enhancements without significant alterations to their overall facial structure. However, when performed alone, it may offer less volume compared with HA fillers. Since it does not directly manipulate the tissues or induce changes in the fascia or skin texture, individuals desiring distinct alterations in facial shape may consider combining it with cog threads. With minimal side effects, situations involving thread protrusion or contact can typically be resolved through gentle removal.
